# Editorial: Proceedings of the 2021 Indiana O'Brien Center Microscopy Workshop

**DOI:** 10.3389/fphys.2022.891526

**Published:** 2022-05-02

**Authors:** Kenneth W. Dunn, Andrew M. Hall, Bruce A. Molitoris

**Affiliations:** ^1^ Division of Nephrology, Indiana University School of Medicine, Indianapolis, IN, United States; ^2^ Institute of Anatomy, University of Zurich, Zurich, Switzerland

**Keywords:** Microscopy, image analysis, tissue cytometry, spatial transcriptomics, machine learning

In June of 2021, the NIH P-30 funded Indiana O’Brien Center for Advanced Renal Microscopic Analysis held its eighth advanced microscopy workshop. Presented every two years since the Center’s inception in 2002, the goal of the workshop is to provide renal investigators with hands-on training and encourage the application of exciting new techniques of microscopy and image analysis to the study of kidney disease. Held in Indianapolis, Indiana in the spring the workshop is designed around a model of 4–5 days of morning lectures followed by afternoon hands-on laboratory exercises in confocal microscopy, multiphoton intravital microscopy and digital image analysis. However, due to concerns about the safety of participants during the Covid-19 pandemic, the workshop was held online in 2021. While the switch precluded the hands-on laboratory experiences that were an important part of previous workshops, the virtual format had a silver lining as it facilitated participation by a broad range of microscopy experts from around the world and allowed for more attendees. As a consequence, the workshop featured lectures and demonstrations from four members of the Indiana O’Brien Center and from a stellar field of 20 leaders in the field of microscopy and was viewed by hundreds of attendees from around the world.

The meeting proceedings are presented in this volume of Frontiers in Physiology. Each day had a unique theme underscoring new developments, and their applications, in areas critical for the advancement of microscopy. Specifically designed to catalyze interactions between the four speakers of each day, the meeting offered a unique view of the evolving fields and input into utilization, challenges and future advances expected. Each presenter was asked to contribute a mini-review to this issue and ten agreed.

The first day of the workshop consisted of an overview of the imaging research and development being conducted by the Indiana O’Brien Center. Bruce Molitoris described how intravital multiphoton microscopy has advanced our understanding of kidney physiology and pathophysiology. Tarek Ashkar presented new methods of 3D tissue cytometry that he has developed to elucidate the mechanisms of kidney disease from animal tissues and human biopsies. Michael Eadon introduced novel approaches to spatial transcriptomics, providing unique insights into localized transcriptional responses to kidney injury. Ken Dunn described methods of digital image analysis that the Indiana O’Brien Center has developed to support quantitative studies of living animals and fixed human and animal tissues. Finally, Seth Winfree presented a description and tutorial on the use of the Volumetric Tissue Exploration and Analysis software, a unique software tool that he developed that provides a complete, interactive workflow solution to tissue cytometry. Perspectives on all five of these topics are included in this volume.

The second day of the workshop was devoted to the new field of large-scale tissue cytometry, an exciting new technique capable of providing a census of every cell and its physiological state in millimeter-scale tissue samples. Bernd Bodenmiller (Zurich, Switzerland) and Lloyd Cantley (Yale, United States) presented examples of how they have applied the technique of quantitative imaging mass cytometry to the study of cancer and kidney disease, respectively. Jeffrey Spraggins (Vanderbilt, United States) described how he has combined imaging mass spectrometry, multiplexed fluorescence microscopy and clinical microscopy to characterize the human kidney for the NIH Human Biomolecular Atlas Program. Michael Gerner (Washington, United States) presented a powerful new integrated approach that he has developed, combining methods of tissue clearing, multiplexed fluorescence microscopy and quantitative image analysis to characterize immune cell microenvironments throughout entire organs. Perspectives from Drs. Cantley and Spraggins are included in this volume.

The third day of the workshop was focused on advances in digital image analysis, a critical aspect of biological microscopy that has become increasingly challenging as the scale and complexity of biological microscopy has grown. Carolina Wahlby (Uppsala, Sweden) and Peter Horvath (Szeged, Hungary) presented an overview of the unique challenges of image analysis in biological microscopy and the solutions developed by their laboratories. David van Valen (Caltech, United States) and Pinaki Sarder (Buffalo, United States) described how they have developed and applied methods of machine learning to improve the quality and power of image analysis in research and pathology. Contributions from Drs. Wahlby and Sarder are included in this volume.

The last day of the workshop featured lectures on new approaches to high-content transcriptomic imaging and efficient large-scale imaging achieved by light-sheet microscopy. Joakim Lundeberg (SciLifeLab, Stockholm, Sweden), Jamie Marshall (Broad Inst., United States) and Yodai Takei (Caltech, United States) described how new techniques of spatial transcriptomics can be used to characterize gene transcription in the spatial context of tissues to better understand human disease. Jonathan Liu (Washington, United States) and Denise Marciano (Texas Southwestern, United States) demonstrated how new methods of light-sheet microscopy facilitate the efficient collection of enormous tissue volumes, extending the power of microscopy as a tool in research and pathology. An overview of Dr. Marshall’s presentation is included in this volume.

The unique confluence of imaging experts assembled at the 2021 O’Brien Center Workshop provided participants with an outstanding overview of clever new approaches that have profoundly extended the scale and scope of biological microscopy. They also pointed the way to how we address the challenges to fully realize the potential of this rich data, including software designed for interactive exploration of massive, complex image volumes, methods of automated image analysis supporting reproducible quantitative analysis, and methods of data analysis designed to help researchers explore and detect latent patterns in highly-multiplexed spatial data. Over the course of the workshop, a consistent theme emerged–each of these solutions was developed through a collaboration between experts from disparate, complementary fields, including microscopy, molecular biology, chemistry, computer science, signal processing, machine learning and data analytics. In this sense, the meeting demonstrated the potential of inter-disciplinary science at its best. The editors hope readers find this volume stimulating and look forward to seeing where these collaborations lead in the future.

